# Prognostic stratification based on m^5^C regulators acts as a novel biomarker for immunotherapy in hepatocellular carcinoma

**DOI:** 10.3389/fimmu.2022.951529

**Published:** 2022-09-09

**Authors:** Ping Liu, Ziqing Zhu, Jiayao Ma, Le Wei, Ying Han, Edward Shen, Xiao Tan, Yihong Chen, Changjing Cai, Cao Guo, Yinghui Peng, Yan Gao, Yongting Liu, Qiaoqiao Huang, Le Gao, Yin Li, Zhaohui Jiang, Wantao Wu, Yihan Liu, Shan Zeng, Wei Li, Ziyang Feng, Hong Shen

**Affiliations:** ^1^ Department of Oncology, Xiangya Hospital, Central South University, Changsha, China; ^2^ Key Laboratory for Molecular Radiation Oncology of Hunan Province, Xiangya Hospital, Central South University, Changsha, China; ^3^ Department of Life Science, McMaster University, Hamilton, ON, Canada; ^4^ National Clinical Research Center for Geriatric Disorders, Xiangya Hospital, Central South University, Changsha, China

**Keywords:** m^5^C, immunotherapy, biomarker, drug sensitivity, HCC, precision medicine

## Abstract

**Background:**

Immunotherapy is a promising anti-cancer strategy in hepatocellular carcinoma (HCC). However, a limited number of patients can benefit from it. There are currently no reliable biomarkers available to find the potential beneficiaries. Methylcytosine (m^5^C) is crucial in HCC, but its role in forecasting clinical responses to immunotherapy has not been fully clarified.

**Methods:**

In this study, we analyzed 371 HCC patients from The Cancer Genome Atlas (TCGA) database and investigated the expression of 18 m^5^C regulators. We selected 6 differentially expressed genes (DEGs) to construct a prognostic risk model as well as 2 m^5^C-related diagnostic models.

**Results:**

The 1-, 3-, and 5-year area under the curve (AUC) of m^5^C scores for the overall survival (OS) was 0.781/0.762/0.711, indicating the m^5^C score system had an ideal distinction of prognostic prediction for HCC. The survival analysis showed that patients with high-risk scores present a worse prognosis than the patients with low-risk scores (*p*< 0.0001). We got consistent results in 6 public cohorts and validated them in Xiangya real-world cohort by quantitative real-time PCR and immunohistochemical (IHC) assays. The high-m^5^C score group was predicted to be in an immune evasion state and showed low sensitivity to immunotherapy, but high sensitivity to chemotherapy and potential targeted drugs and agents, such as sepantronium bromide (YM-155), axitinib, vinblastine and docetaxel. Meanwhile, we also constructed two diagnostic models to distinguish HCC tumors from normal liver tissues or liver cirrhosis.

**Conclusion:**

In conclusion, our study helps to early screen HCC patients and select patients who can benefit from immunotherapy. Step forwardly, for the less likely beneficiaries, this study provides them with new potential targeted drugs and agents for choice to improve their prognosis.

## Introduction

Primary liver cancer is a common gastrointestinal malignancy and the fourth leading cause of cancer-related death worldwide, with approximately 782,000 deaths each year (1). HCC is the most prevalent subtype of primary liver cancer, as the 5-year overall survival rate is less than 20% (2, 3).

The recent achievement of immune checkpoint blockers (ICBs) has established immunotherapy as the most promising cancer treatment strategy. Despite the advancement achieved in therapeutic modalities for HCC, the overall survival (OS) remains poor (4). The reason is that many patients are diagnosed at an advanced stage, an abundance of HCC patients exhibit hyposensitivity to these therapies, and the nearly unavoidable drug resistance stands in the way of an eventual cure (5). There are currently no reliable and validated biomarkers or methods available to early screen the HCC patients and correctly forecast clinical responses to immunotherapy. Therefore, the development of meaningful diagnosis and treatment response stratification biomarkers for achieving precision medicine is urgently needed.

The TME is a heterogeneous system that is comprised of cancer cells, immune cells, extracellular matrix, microvessels, and a variety of cytokines and chemokines (6, 7). It plays an important role in the pathogenesis of HCC (8). Patients with the same pathological stage and grade have considerably diverse functional hallmarks, which might lead to variable clinical responses to the same therapy. In particular, substantial TME heterogeneity makes precision medicine in HCC difficult to obtain. Thus, displaying TME heterogeneity might disclose several aspects of HCC biology and expand our understanding of HCC therapy. In the setting of TME heterogeneity, developing innovative therapeutic response predictors and therapeutic targets could be a prospective way to promote precision medicine in HCC.

According to etiological research, a range of environmental stresses generates changes in liver RNA, which eventually bring about changes in the liver epigenome and transcriptome, which also implies that epigenetic modifications accelerate the onset and development of HCC (9, 10). Exploring the role of RNA modification in a range of biological processes has recently emerged as a new research focus (11). The most common modifications currently include N6-methyladenosine (m^6^A), m^5^C, 5-hydroxymethylcytosine (hm^5^C), N7-methylguanosine (m^7^G), N1-methyladenosine (m^1^A), and pseudouridine (ψ) (12). The role of m6A modification in regulating RNA processing and functioning has been studied extensively in the past (13). Evolving data suggest that m^5^C plays a significant part in posttranscriptional regulation (14). Furthermore, m^5^C modification, characterized by the insertion of a methyl group at the carbon-5 position of the cytosine base, was discovered to be copious in mammalian cells (15). m^5^C regulation is a dynamic process governed by three key regulators, methyltransferases (writers), demethylases (erasers), and binding proteins (readers) (16). Increasing data have demonstrated that m^5^C modification emerges as a contributor to shaping TME heterogeneity, as well as the progression of HCC through interaction with various m^5^C regulators (14). In addition, there is growing evidence that methylation regulators can serve as prognostic and diagnostic markers for cancer (17). A better knowledge of different m^5^C modification patterns in HCC would contribute to the analysis of HCC diagnosis and prognosis, thus guiding individual clinical diagnosis and treatment strategies.

The past research on m^5^C regulators-mediated methylation modification model in HCC was focused on the effect of a single TME cell type or single m^5^C regulator alone on tumor development, and the overall TME infiltration portrait mediated by several m^5^C regulators remains to be comprehensively recognized. In addition, present predictive models are limited to predicting the prognosis and the immunotherapy response but overlook the diagnostic role. And to better achieve precision medicine, more attention should be paid to patients who are less susceptible to immunotherapy. Moreover, existing researches lack real-world cohort validation and the selected regulators are incomprehensive (18, 19).

**Figure 1:**
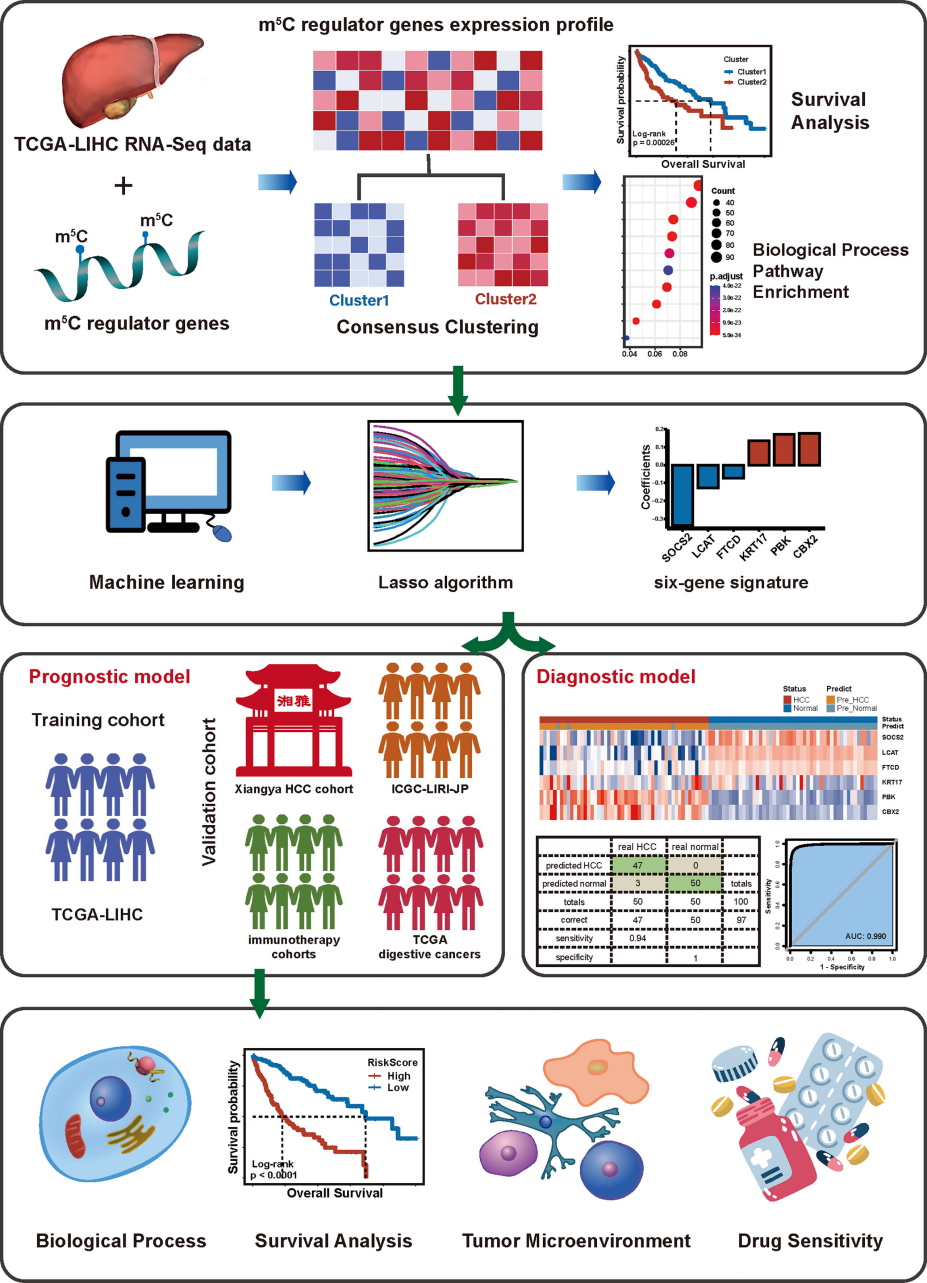
The workflow of our study.

As a result, we took the mRNA expression levels of 18 m^5^C regulators into consideration to assess their comprehensive relevance to diagnosis, TME heterogeneity, drug sensitivity, and therapeutic opportunities and identified robust risk signatures for HCC prognosis. Then, we (I) constructed 2 diagnostic and a prognostic model based on comprehensive 18 m^5^C regulators; (II) built an m^5^C score to predict immunotherapy response, and (III) determined potential drugs in HCC (**Figure 1**).

## Methods and materials

### Data collection and processing

The TCGA data included TCGA-LIHC and other TCGA digestive cancers (TCGA-COAD, TCGA-READ, TCGA-STAD, TCGA-CHOL, and TCGA-PAAD) with clinical information were obtained from the Genomic Data Commons (GDC) Data Portal (https://portal.gdc.cancer.gov/). ICGC-LIRI-JP datasets were downloaded from the International Cancer Genome Consortium (ICGC) Data Portal (https://dcc.icgc.org/). GSE14520 datasets were obtained from Gene Expression Omnibus databases (GEO). The IMvigor210 cohort (20) data were obtained from the “IMvigor210CoreBiologies” R package. Riaz et al. Cell 2017 (GSE91061) (21) and Lauss et al. Nat Commun 2017 (GSE100797) (22) were gathered from the TIDE website (http://tide.dfci.harvard.edu/) with a detailed clinical information and gene expression data. The transcripts per kilobase million (TPM) values of gene expression were used for further analysis.

### Unsupervised clustering for 18 m^5^C regulators

Sum up to 18 m^5^C regulators were selected to identify different m^5^C clusters by m^5^C regulators. Based on the gene expression data of these m^5^C regulators in the training cohort (TCGA-LIHC cohort), we used an unsupervised cluster algorithm *via* the R package “ConsensuClusterPlus”. The R package “NbClust” and Silhouette algorithm were applied to verify the optimal cluster number further. Principal component analysis (PCA) and tSNE were used to visualize different clusters in two-dimensional.

### Identifying DEGs between m^5^C cluster

We distinguished the DEGs between two m^5^C clusters *via* the “DESeq2” R package. The DEGs were defined as adjusted *p*-value< 0.05 and |log2FoldChange| > 1. A total of 1267 DEGs between two m^5^C clusters were extracted for further analysis.

### Functional enrichment for DEGs

To explore the functions of DEGs, enrichment analysis of these genes was conducted by R package “clusterprofiler” R package, based on the Kyoto Encyclopedia of Genes and Genomes (KEGG) and Gene Ontology (GO) databases. False discovery rate (FDR)< 0.25 and *p*-value< 0.05 were considered statistically significant.

### Gene set variation analysis

To investigate the differential pathway of m^5^C clusters, the gene set variation analysis “GSVA” R package was used to perform GSVA. The gene sets of hallmarks were downloaded from the MSigDB database for GSVA analysis. The adjusted *p*-value< 0.05 was considered statistically significant.

### Gene signature identification and establishment of the prognostic risk model

We performed the prognostic analysis for DEGs in two m^5^C clusters using a univariate Cox regression. As a result, 142 DEGs with a significant prognosis while a *p*-value ≤ 0.0001 were extracted for further analysis. The prognostic risk model was constructed by using the “glmnet” and “My.stepwise” R package based on LASSO and stepwise Cox method. Finally, we obtained a 6-gene-based prognostic risk model to calculate each patient’s risk score by weighting the Cox regression coefficients. The formula is as follows:


$$RiskScore=\;\underset{i}{\sum^{}}Coef_{i}*\;expr_{i}$$

RiskScore = coefficient of each modeled gene * expression of each modeled gene, where “ i “ represents the modeled gene, “ Coef “ represents the coefficients of regression, and “ expr “ represents the expression of gene.We verified the prognostic risk model in validation cohorts. The time-dependent receiver operating characteristic (ROC) curves were used to evaluate the prognostic prediction accuracy of the risk model and the area under the curve (AUC) was measured by the R package “survivalROC”.

### Estimation of TME characterization

To explore the correlation between the prognostic risk model and the immune cell infiltration level, we use the CIBERSORT algorithm to assess the infiltration abundance of 22 immune cells. And we obtained collected 20 inhibitory immune checkpoints from Auslander’s study (23). The Tumor Immune Dysfunction and Exclusion (TIDE) and IPS algorithms were used to evaluate tumor immune escape status. To compare the TME characterization of two different clusters, first, the R package “IOBR” was used to analyze the TME pathway *via* the single-sample gene set enrichment analysis (ssGSEA) (24, 25) algorithm. Meanwhile, a set of gene signatures that represent a non-inflamed TME, and tumor therapy-associated response were collected from Jiao Hu’s study (26).

### Drug sensitivity and potential compounds

Broad Institute-Cancer Cell Line Encyclopedia (CCLE) project (https://portals.broadinstitute.org/ccle/) contained 1019 cancer cell line RNA expression profile data. Drug sensitivity data of cancer cell lines were obtained from the Genomics of Drug Sensitivity in Cancer (GDSC2, https://www.cancerrxgene.org/) and the PRISM Repurposing dataset (19Q4, released December 2019, https://depmap.org/portal/prism/). The GDSC2 contains 809 cell lines and sensitivity data for 198 compounds, and the secondary PRISM contains 499 cell lines and drug susceptibility data for 1448 compounds. All three of these drug datasets provide the area under the dose-response curve (area under the curve — AUC) values as a measure of drug susceptibility, and lower AUC values indicate increased susceptibility to compound responses. The drug susceptibility of the TCGA-LIHC cohort was estimated *via* the R package “oncoPredict”.

Connectivity Map (CMap) analysis was a complement to further investigate the therapeutic potential of drugs in HCC. We first analyzed differential expression genes between the TCGA-LIHC tumor and normal samples. And 150 up-regulated genes and 150 down-regulated genes were submitted to the CMap website (https://clue.io/query). The CMap analysis yielded a connectivity score for each perturbation, a negative score represents a gene expression pattern of a certain perturbation that is oppositional to the disease-specific expression pattern, suggesting a potential therapeutic effect of this perturbation in this disease.

### Xiangya cohort

55 fresh hepatocellular carcinoma tissues and part of paired normal liver tissues were collected from Xiangya hospital and immediately frozen in liquid nitrogen. A retrospective cohort was created, with a total of 55 patients enrolled as of September 1, 2021. After the follow-up period, the clinical characteristics were obtained, and survival analysis and multivariate Cox regression were performed. This study was reviewed and approved by the Xiangya Hospital Medical Ethics Committee of Central South University (No.201806928), and got consent from all participants.

### Quantitative reverse-transcription PCR

The AG RNAex Pro Reagent (AG21102, Accurate Biology, Changsha, China) was used to extract total RNA from 55 fresh hepatocellular carcinoma tissues and part of paired normal liver tissues according to the manufacturer’s instructions. Before reverse transcription to cDNA, genomic DNA is eliminated by treatment for 2 min at 42°C with gDNA Clean Reagent. Evo M-MLV RT Kit (AG11705, Accurate Biology, Changsha, China) was used to synthesize the complementary RNA. The SYBRR Premix Pro Taq HS qPCR Kit (AG11708, Accurate Biology, Changsha, China) was utilized to achieve real-time quantification. The 2^-△△CT^ approach was applied to assess the relative expression levels of target genes, which were normalized by GAPDH. The PCR primers are listed in **Supplementary Table S6**.

### Immunohistochemical staining

The protein expression levels of Programmed death-ligand 1 (PD-L1; also called B7-H1 or CD274) and cytotoxic T-lymphocyte antigen-4 (CTLA4) were estimated *via* immunohistochemical (IHC) staining. Briefly, after deparaffinization and rehydration, antigen retrieval was performed by heating the slides in EDTA buffer (G1203; Servicebio, Wuhan, China). Then the slides were treated with 3% hydrogen peroxide (Annjet, Shandong, China) for 25 mins to eliminate endogenous peroxidase and blocked with 3% BSA (G5001; Servicebio) for 30 minutes at room temperature to decrease nonspecific binding. The slides were then incubated overnight at 4°C with rabbit anti-CD274 (PDL1) and anti-CTLA4 primary antibodies at a dilution of 1:200 and 1:300, respectively (CD274: catalog no. Ab205921, Abcam, UK; CTLA4: catalog no. ab237712, Abcam, UK). Next, the slides were incubated with Goat Anti-rabbit IgG/HRP secondary antibody (G1215; Servicebio) for 50 minutes at 37°C. The DAB solution was used for coloration, and hematoxylin was used for counterstaining.

Immunostaining of CD274 (PDL1) and CTLA4 were defined on a scale of 0 to 3 in which 0 means no staining, 1 means mild staining, 2 means medium staining, and 3 means intense staining. The percentage score of stained cells were also calculated on a scale of 1 to 4 in which 1 represents (0–25%), 2 = (26–50%), 3 = (51–75%) and 4 = (76– 100%). To obtain the final score, the intensity score and percentage score were added to reach the final score ranging from 0 to 7.

### Statistical analysis

Correlations between variables were explored with Pearson correlation coefficients. Continuous variables were compared between two groups through the Wilcoxon rank-sum test. Survival analysis including Kaplan-Meier and Cox regression analysis was performed by “survival” R package. The optimal cut-off value in training cohort TCGA-LIHC and validation cohorts were determined by the “surv_cutpoint” function in the “survminer” R package. Unless otherwise stated, *P-value*< 0.05 was regarded as statistically significant. The whole work was conducted in R 4.1.2 software.

## Result

### The landscape of expression and genetic variation of m^5^C regulators in HCC

**Figure 2:**
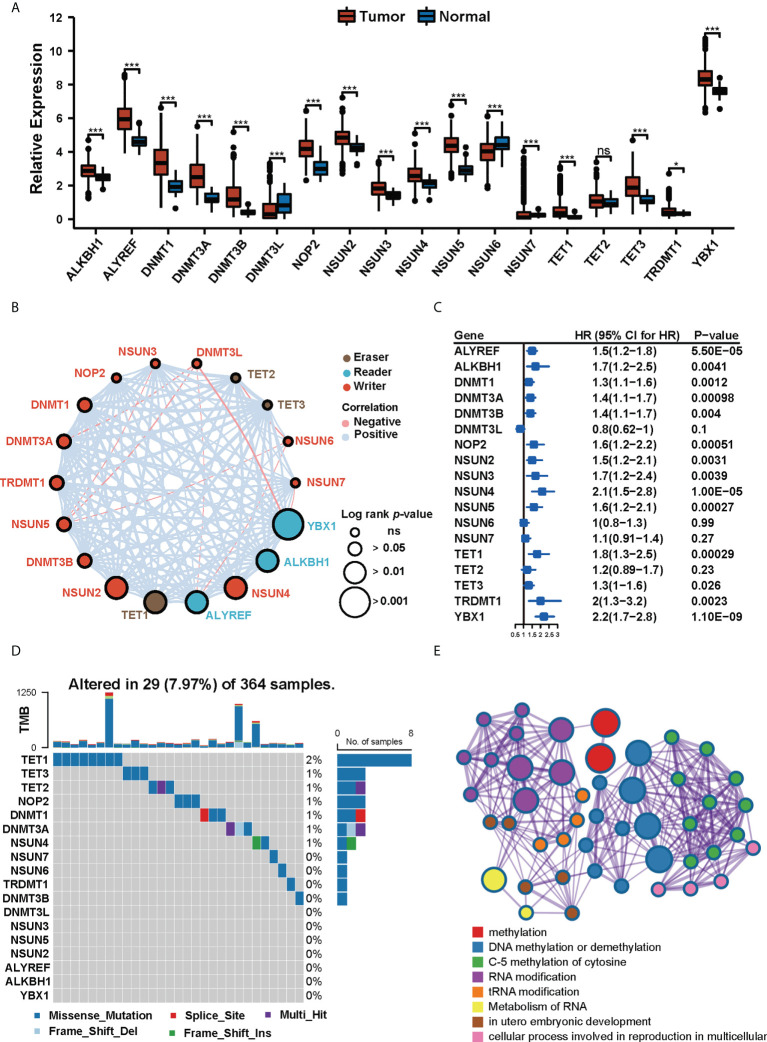
The landscape of expression and genetic variation of m^5^C regulators in HCC. **(A)** The expression profiles of m^5^C regulator genes in tumor tissues and normal tissues in TCGA-LIHC cohort. (* p<0.05, *** p<0.001, ns: no significant difference). **(B)** The interactions of 18 m^5^C regulator genes and their prognostic value. **(C)** Univariate Cox regression analysis of the 18 m^5^C regulator genes in patients from TCGA-LIHC cohort. **(D)** The mutation frequency of 18 m^5^C regulators in 364 patients from the TCGA-LIHC cohort. **(E)** Metascape enrichment network visualization of 18 m^5^C regulators. Cluster annotations were shown in the color code.

We collected 18 m^5^C regulator genes from previous studies (27–31). The multi-omics landscape of these 18 m^5^C regulator genes was summarized from the TCGA-LIHC cohort (**Figure 2**). Most of the m^5^C regulator genes were significantly differentially expressed in tumor and normal tissues. For instance, ALKBH1, ALYREF, DNMT1, DNMT3A, DNMT3B, NOP2, NSUN2, NSUN3, NSUN4, NSUN5, TET1, TET2, TET3, TRDMT1, and YBX1 were up-regulated in HCC, while DNMT3L, NSUN6, and NSUN7 were down-regulated (**Figure 2A**). We then explored the comprehensive correlations and prognostic value of m^5^C regulators in the TCGA-LIHC cohort (**Figure 2B**). The univariate Cox regression analysis further demonstrated that most of these m^5^C regulator genes played a risk factor role in HCC (**Figure 2C**). Then we found out that these genes had widespread copy number variations (CNVs) (**Figure S1**) but infrequent mutations (**Figure 2D**). For example, ALYREF, DNMT3A, DNMT3B, DNMT3L, NOP2, NSUN2, NSUN4, NSUN5, NSUN6, NSUN7, TET1, and TRDMT1 focused on copy number amplification, whereas ALKBH1, DNMT1, TET2, and YBX1 preferred deletion, which suggests that CNVs’ dominant role in the regulation of m^5^C relative to mutation. Metascape (32) is a web tool for gene annotation and analysis, the enrichment analyses of 18 m^5^C regulators are displayed in **Figure 2E**.

### Clustering of HCC based on 18 m^5^C regulators

A total of 371 samples from TCGA-LIHC were defined as the training cohort, and further divided into k clusters (k = 2~6) *via* the “ConsensusClusterPlus” R package. We found out that k = 2 was the best number of clusters according to the CDF curve of the consensus score (**Figures S2A, B**). The Silhouette algorithm and “NbClust” R package further confirmed the result (**Figures S2C, D**). The principal component analysis and tSNE method of 18 m^5^C regulator gene expression showed significant separation between two clusters (**Figures S2E, F**). Finally, 248 patients were classified as Cluster 1, and the rest 123 patients were classified as Cluster 2. Compared to Cluster 1, the Kaplan-Meier analysis showed a worse OS and progression-free survival (PFS) in Cluster 2 (**Figures S2G–H**, log-rank test, *p*< 0.001).

### Clinical characteristics of gene set enrichment between clusters

**Figure 3:**
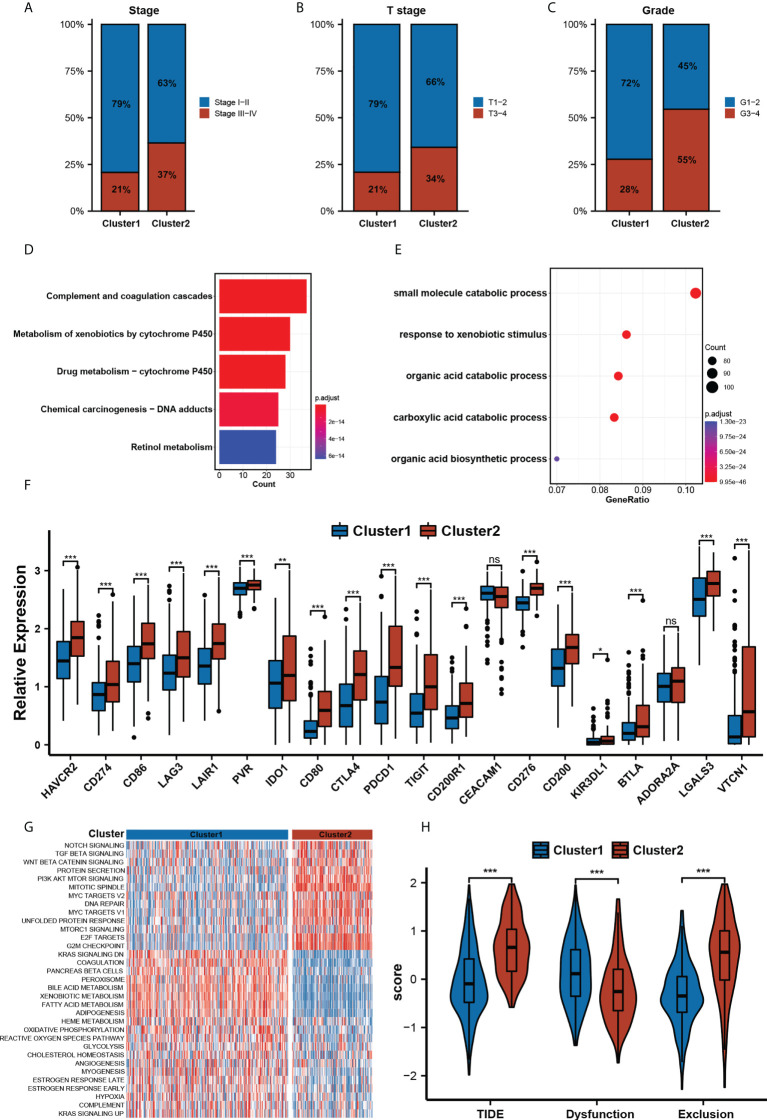
Clinical Characteristics Gene Sets Enrichment between clusters. **(A-C)** The clinical characteristics included Neoplasm Disease Stage. **(A)**, Cancer Tumor Stage **(B)**, and Neoplasm Histologic Grade **(C)** between two m^5^C clusters(Chi-square test, p< 0.01). **(D, E)** The top 10 significant GO analysis terms **(D)** and KEGG pathways **(E)** of DEGs in two clusters. **(F)**. The relative expression levels of 20 immune checkpoints in two m^5^C clusters. (* p<0.05, ** p<0.01, *** p<0.001, ns: no significant difference). **(G)**. HALLMARK pathway enrichment analysis of two m^5^C clusters by GSVA. **(H)**. The relative distribution of TIDE was compared between two m^5^C clusters.

To further explore the differences between the two clusters, we compared the clinical characteristics between the two clusters. The result obtained shows that showed Cluster 2 had a higher proportion of advanced disease stage, tumor stage, and histologic grade (**Figures 3A–C**, Fisher’s exact test, *p<* 0.01). We then analyzed the differentially expressed genes (DEGs) by using the “DESeq2” R package, a total of 1267 DEGs between two m^5^C clusters were obtained according to the adjusted *p*-value less than 0.05, and the absolute value of log2 fold change less than 1. Next, these DEGs were used for biological functional enrichment analysis. These DEGs were primarily enriched in stromal and catabolic as well as metabolic pathways (**Figures 3D, E**), such as small molecule catabolic process (GO term), response to xenobiotic stimulus (GO term), organic acid catabolic process (GO term), complement and coagulation cascades (KEGG pathway), metabolism of xenobiotics by cytochrome P450 (KEGG pathway), and drug metabolism - cytochrome P450 (KEGG pathway). Further GSVA analysis on hallmark pathway analysis (**Figure 3G**) revealed that patients in Cluster 2 exhibited an obvious enrichment of pathways involved in cell cycle, DNA repair, and carcinogenic activation pathways for example WNT-β-Catenin signaling, TGF-β signaling, PI3K-AKT-MTOR signaling, MYC targets, etc. Moreover, we found out that Cluster 2 had a higher expression of immune checkpoints than Cluster 1 (**Figure 3F**). Our analysis also revealed that Cluster 2 had a significantly increased TIDE score and Exclusion score but decreased Dysfunction score (**Figure 3H**), which suggests that these patients in Cluster 2 may have an immune evasion mechanism.

### Construction of the prognostic risk model

**Figure 4:**
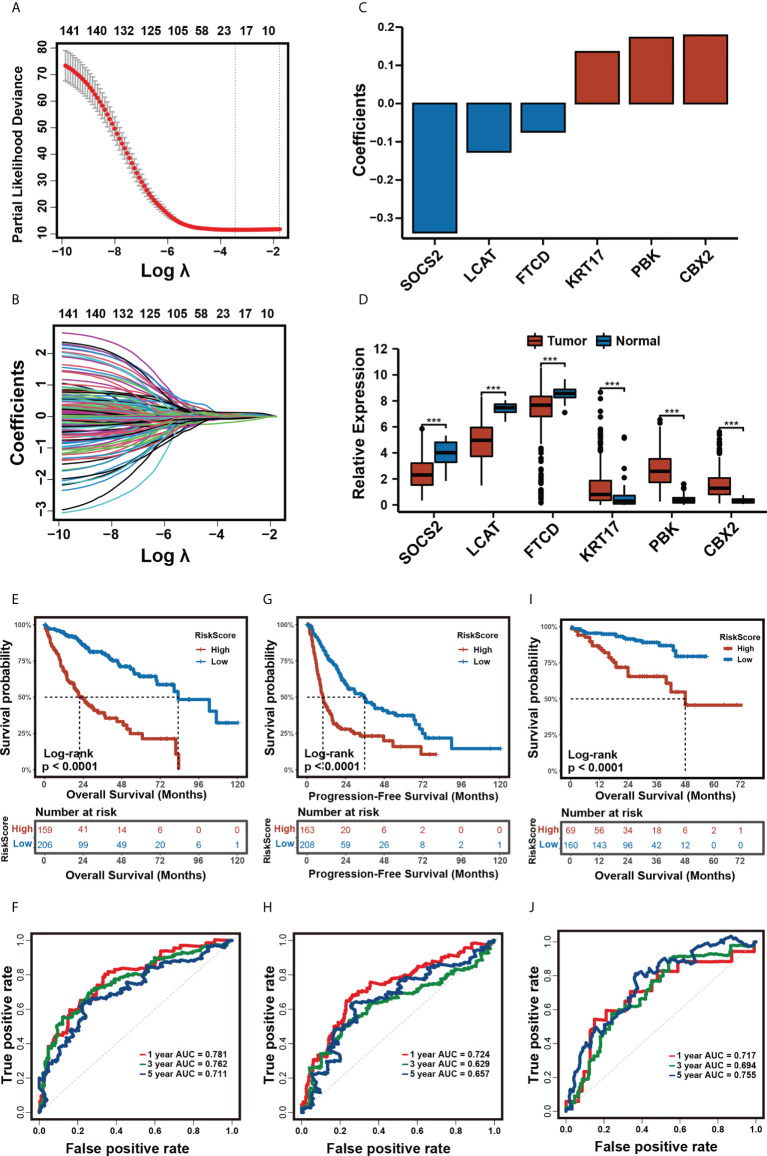
Construction of the prognostic risk model. **(A, B)** The least absolute shrinkage and selection operator (LASSO) regression was performed, calculating the minimum criteria. **(C)**. Coefficients plot of six selected genes. **(D)**. Differential expression of six selected genes in tumor and normal tissues in TCGA-LIHC cohort. (*** p<0.001). **(E, F)** Kaplan-Meier analysis for OS **(E) **and time-dependent ROC curve **(F)** of the risk score in the TCGA-LIHC cohort. **(G, H)** Kaplan-Meier analysis for DFS **(G)** and time-dependent ROC curve **(H)** of the risk score in the TCGA-LIHC cohort. **(I, J)** Kaplan-Meier analysis for OS **(I)** and time-dependent ROC curve **(J)** of the risk score in the ICGC cohort.

We constructed a prognostic risk model to quantify the risk for each patient based on our m^5^C clustering. After univariate Cox regression, 142 DEGs with a significant prognosis at a *p*-value ≤ 0.0001 were analyzed by the LASSO regression algorithm in the TCGA-LIHC cohort (**Figures 4A, B**). In stepwise variable selection procedures, the Cox regression algorithm was used to filter the variables further. Finally, a total of 6 candidate genes including CBX2, SOCS2, LCAT, PBK, KRT17, and FTCD (**Figures 4C, D**) were selected to construct the prognostic risk model. The patients in the training cohort TCGA-LIHC were separated into low- and high-m^5^C score groups by the optimal cut-off value calculated by the “surv_cutpoint” function in the “survminer” R package. In the training cohort, TCGA-LIHC (**Table S1**), the Kaplan–Meier analysis revealed that the high-m^5^C score group had a significantly worse OS and PFS than the low-m^5^C score group (**Figures 4E, G**). Multivariate Cox analysis of the training cohort revealed that the m^5^C score can be an independent prognostic factor (**Figure S3**). The 1-, 3-, and 5-year AUC of m^5^C scores for OS was 0.781/0.762/0.711 (**Figure 4F**), and for PFS, the 1-, 3-, and 5-year AUC was 0.724/0.629/0.657 (**Figure 4H**). Likewise, in the validation cohorts ICGC-LIRI-JP and GSE14520, our risk model still had a stable prognostic capability, the high-m^5^C score patients manifested a significantly worse OS than the low-m^5^C score patients (**Figure 4I**, **Figure S4A**), and the 1-, 3-, and 5-year AUC were 0.717/0.694/0.755 and 0.619/0.622/0.627 (**Figure 4J** and **Figure S4B**).

### Assessment of immune microenvironment characterization with the prognostic risk model

**Figure 5:**
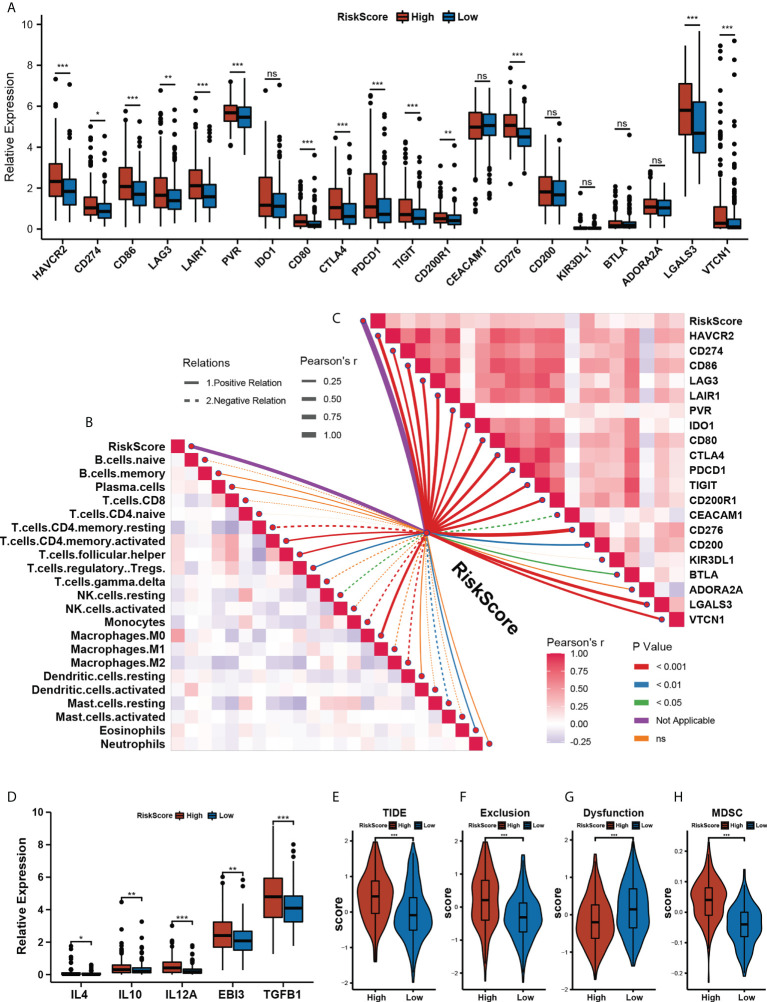
Assessing immune microenvironment characterization with the prognostic risk model. **(A)** The relative expression levels of 20 immune checkpoints in the High- and Low- m^5^C score group.(* p<0.05, ** p<0.01, *** p<0.001, ns: no significant difference). **(B)** The correlations between the m^5^C score and immune cell infiltration were estimated by using the CIBERSORT algorithm. **(C)** The correlations between the m^5^C score and immune checkpoints expression. **(D)** Immunoinhibitory cytokines expression between High- and Low- m5C score groups. **(E–H)** TIDE **(E)** Exclusion **(F)** Dysfunction **(G)** and MDSC score between High- and Low- m^5^C score groups. **(H)** Relative distribution of tumor mutation load in High- versus Low- m5C score groups. (* p<0.05, ** p<0.01, *** p<0.001, ns: no significant difference).

To investigate the role of our risk model in the immune microenvironment of HCC, we looked into the correlation among the m^5^C score and expression level of immune checkpoints, immune cells infiltration, TIDE, and IPS scores in the training cohort TCGA-LIHC. The relative expression of most of the 20 immune checkpoints was significantly elevated in the high- m^5^C score group (**Figure 5A**). To further analyze the correlations between the immune microenvironment and our prognostic risk model, we used the CIBERSORT algorithm to calculate 22 types of immune cells, **Figure 5B** shows the correlation between the m^5^C score and immune infiltration cells. The m^5^C score displayed a significantly positive correlation with macrophages (M0), activated memory CD4 T cells, follicular helper T cells, and Eosinophils (*p*< 0.01), and a significantly negative correlation with resting memory CD4 T cells, resting NK cells, monocytes, macrophages (M2), and resting mast cells (*p*< 0.01). Not only CIBERSORT, but we also used TIMER and MCPcounter to analyze immune cell infiltration in high and low-risk groups, the result were displayed in **Figure S5**. Additionally, we also analyzed the correlation between the expression of immune checkpoints and our risk model score. Most of the immune checkpoints had a significantly positive correlation with the m^5^C score (**Figure 5C**). We hypothesized that the m^5^C score might be related to immune evasion mechanisms, so we compared the TIDE and IPS scores between the low- and high- m^5^C score groups. The patient in the high-m5C score group had a higher inhibitory immune checkpoints expression level, TIDE and Exclusion score, and a lower Dysfunction score (**Figuress 5D–G**, *p*< 0.001). Myeloid-derived suppressor cells (MDSC) (33) are a group of cells defined by their T cell immunosuppressive functions. We found out that the high-m^5^C score group also presented a higher MDSC score (**Figure 5H**, *p*< 0.001), which further suggests that the high-m^5^C score group may be in an immunosuppressed state. IPS was significantly elevated in the low-m^5^C score group (**Figure S5**, *p*< 0.001). These findings indirectly demonstrate that our risk model had a valuable relevance in the immune microenvironment of HCC.

**Figure 6:**
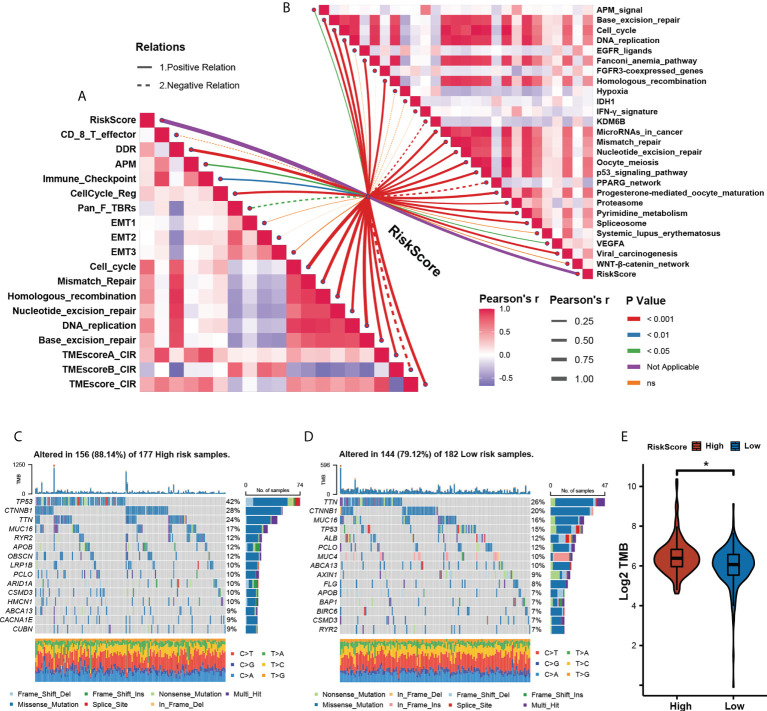
Assessing tumor microenvironment characterization with the prognostic. **(A)**. The correlations between the m5C score and the enrichment scores of TME pathways. **(B)** The correlations between the m5C score and the enrichment scores of immunotherapy- predicted pathways. **(C, D)** The mutational landscape between High- **(C)** and Low- **(D)** m5C score groups. **(E)** Relative distribution of tumor mutation load in High- versus Low- m5C score groups. (* p<0.05).

To further explore the correlation between TME and our risk model, we also analyzed the TME pathway and several therapeutic signatures with our m^5^C score. The result obtained shows a significant positive correlation between the m^5^C score and most TME and therapeutic pathways (**Figures 6A, B**). Growing evidence has displayed an association between somatic mutations in tumor genomes and response to immunotherapy (34–36). We found that though there was no notable difference in the total mutation frequency between the two m^5^C score groups, the high-m^5^C score group exhibited more TP53 mutation than the low-m^5^C score group (42% versus 15%, Fisher’s exact test, *p*< 0.001) (**Figures 6C, D**). Recent research has revealed that TMB may act as a biomarker of response to immune checkpoint inhibitors (37–40). We then compared the TMB between the two m^5^C score groups and found out that the high-m^5^C score group had a higher TMB than the low-m^5^C score group (*p*< 0.05) (**Figure 6E**).

### Assessing the association of potential drug sensitivity with the prognostic risk model

**Figure 7:**
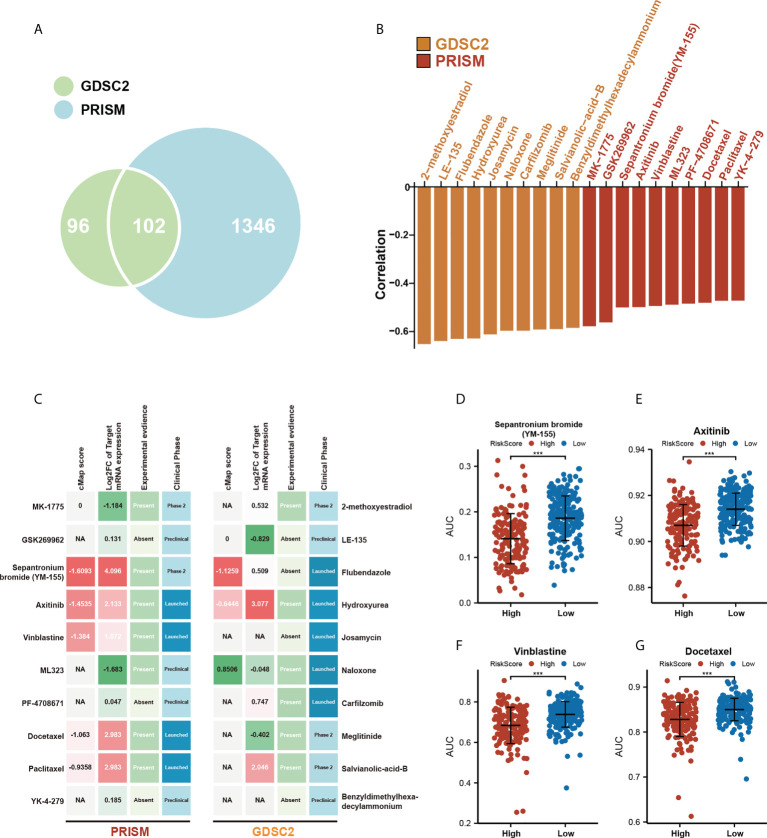
Assessing the association of potential drug sensitivity with the prognostic risk model. **(A)** Venn diagram for summarizing included compounds from GDSC and PRISM datasets. **(B)** Top 10 associated compounds with m^5^C score in two drug databases (GDSC2, PRISM). **(C)** Identification of the most promising therapeutic agents for high m5C score patients according to the evidence from multiple sources, 10 GDSC-derived agents and 10 PRISM-derived agents were shown on the left and right of the diagram, respectively. **(D-G)** Differential drug response AUC analysis of 4 selected compounds (Wilcoxon test, *** *p*< 0.001).

To extend our model for clinical translation, we further explored the possible association of the m^5^C score with potential drug sensitivity. We performed analyses of GDSC2 and PRISM drug sensitivity databases in an attempt to find new potential compounds associated with the m^5^C score (**Figure 7A**). By correlation analysis, we screened the compounds in these two drug sensitivity databases that were negatively correlated with m^5^C scores, and the absolute value of the Pearson’s correlation coefficient obtained > 0.3. Also a significantly different AUC (Wilcoxon test, *p*< 0.05) was observed between the two groups of high- and low-m^5^C scores (**Tables S2**, **S3**). The top 10 associated compounds with m^5^C scores in two drug databases are presented in **Figure 7B**. Although these 20 candidate compounds showed increased drug sensitivity in patients with higher m^5^C scores, the above analyses alone can not lead to the conclusion that these chemicals had a therapeutic effect on HCC. Thus, we used the CMap analysis to find compounds in which gene expression patterns were oppositional to the HCC-specific expression patterns. If CMap scores< −1, it represents that these compounds might bring therapeutic benefits to HCC. Secondly, we calculated fold-change differences in the mRNA expression levels of candidates’ drug target genes between tumor and normal tissue, and a higher fold change value indicated a greater potential for a candidate agent for HCC treatment. Finally, a comprehensive literature search was performed in PubMed to find out the experimental and clinical evidence of candidate compounds for treating HCC. All results were presented in **Figure 7C** (**Table S4**). Four compounds, including Sepantronium bromide (YM-155), axitinib, vinblastine, and docetaxel, which had strong *in vitro* and silico evidence, were thought to be the most promising therapeutic candidates in HCC patients with high m^5^C scores. Differences in the sensitivity of these four drugs between the high- and low-m^5^C score groups were displayed in **Figure 7D-G**. Our results can provide some reference for further clinical translation.

### Validation of risk score in IMvigor210 cohort and other TCGA digestive cancers

To validate the feasibility of our prognostic risk model in immunotherapy and other GI tumors, we applied our model to three immunotherapy cohorts that included IMvigor210, Riaz, et al. Cell 2017 (GSE91061), and Lauss et al. Nat Commun 2017 (GSE100797), and the other five additional TCGA digestive cancer cohorts that also included TCGA-CHOL, TCGA-PAAD, TCGA-STAD, TCGA-COAD, and TCGA-READ. We then found out that our prognostic risk model can distinguish between the high and low-risk groups with a completely different OS or PFS in three immunotherapy cohorts, and TCGA-CHOL, and TCGA-PAAD cohort (**Figures S6A–E**, log-rank test, *p*< 0.05), but showed no significant difference in TCGA-STAD, TCGA-COAD, and TCGA-READ (**Figures S6F–H**) cohorts.

### Constructing diagnostic models by six candidate genes

**Figure 8:**
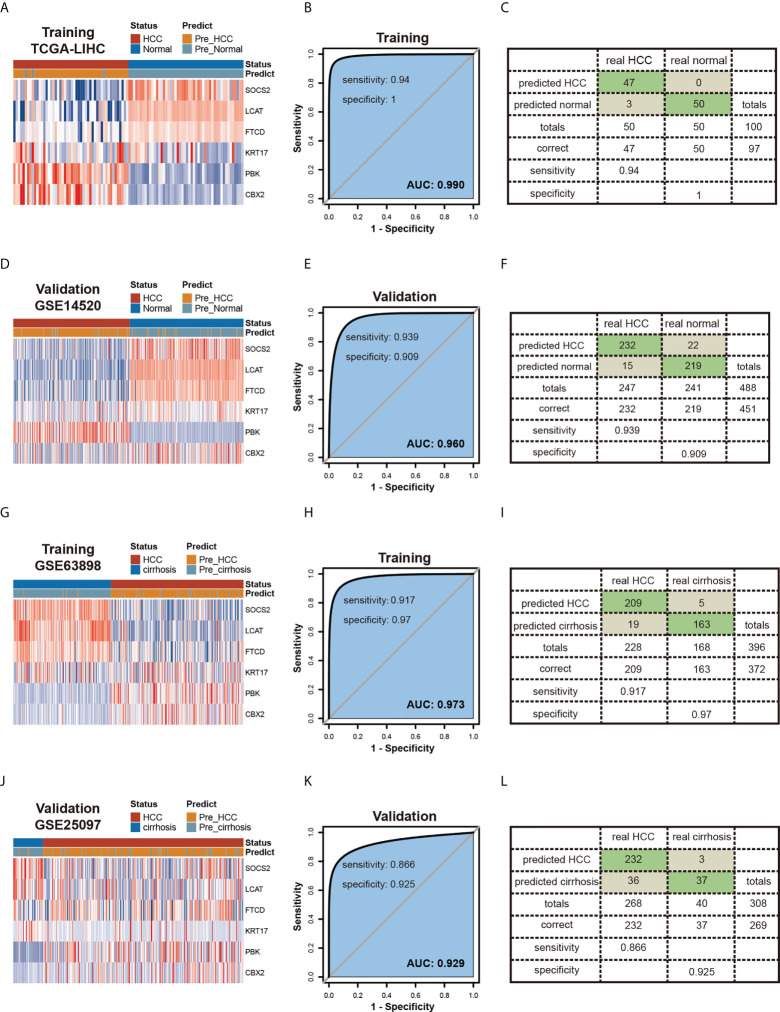
Constructing Diagnostic Models by six candidate genes. **(A-F)** Diagnostic model of HCC and normal tissues.Heatmaps of six genes expression between HCC and normal tissues in the training **(A)** and validation **(D)** testing cohorts.Receiver operating characteristic (ROC) curves and the associated areas under curves (AUCs) of the diagnostic prediction models in the training **(B)** and validation **(E)** testing cohorts.Confusion matrices were built from the diagnostic model prediction in the training **(C)** and validation **(F)** testing cohorts. **(G-L)** Diagnostic model of HCC and cirrhosis tissues.Heatmaps of six genes expression between HCC and cirrhosis tissues in the training **(G)** and validation **(J)** cohorts.Receiver operating characteristic (ROC) curves and the associated areas under curves (AUCs) of the diagnostic prediction models in the training **(H)** and validation **(K)** cohorts. Confusion matrices were built from the diagnostic model prediction in the training **(I)** and validation **(L)** cohorts.

To increase the probability of early detection of HCC, we developed a tumor diagnostic model by these six genes of our prognostic risk model. 50 HCC tumor samples and 50 paired normal tissues from TGCA-LIHC as the training cohort, 247 HCC tumor samples and 241 normal tissues from GSE14520 as the validation cohort to verify the reliability of the model. The tumor diagnostic model was formulated as follows: logit (P-HCC) = 6.906 - 0.494 * SOCS2 expression level - 1.250 * LCAT expression level – 0.493 * FTCD expression level + 0.602 * KRT17 expression level + 1.950 * PBK expression level + 7.243 * CBX2 expression level. The results obtained are shown in **Figures 8A–F**. The AUCs of the training cohort and the validation cohort are 0.99 (**Figure 8B**) and 0.960 (**Figure 8E**), the diagnostic model achieved 94% sensitivity and 100% specificity in the training cohort (**Figure 8C**) and 93.9% sensitivity and 90.9% specificity in the validation cohort (**Figure 8F**). Meanwhile, we also constructed 2 diagnostic models of HCC tumors and liver cirrhosis. The training cohort GSE63898 contained 228 HCC samples and 168 liver cirrhosis samples, and the validation cohort GSE25097 contained 268 HCC samples and 40 liver cirrhosis samples. The tumor diagnostic model was formulated as follows: logit (P-HCC) = -2.534 – 1.240 * SOCS2 expression level - 1.320 * LCAT expression level + 0.335 * FTCD expression level – 1.911 * KRT17 expression level + 4.422 * PBK expression level + 2.006 * CBX2 expression level. The results obtained are shown in **Figures 8G–L**. The AUCs of the training cohort and the validation cohort are 0.973 (**Figure 8H**) and 0.929 (**Figure 8K**), the diagnostic model achieved 91.7% sensitivity and 97.0% specificity in the training cohort (**Figure 8I**) and 86.6% sensitivity and 92.5% specificity in the validation cohort (**Figure 8L**).

### Validation of the prognostic risk model in Xiangya HCC cohort

**Figure 9:**
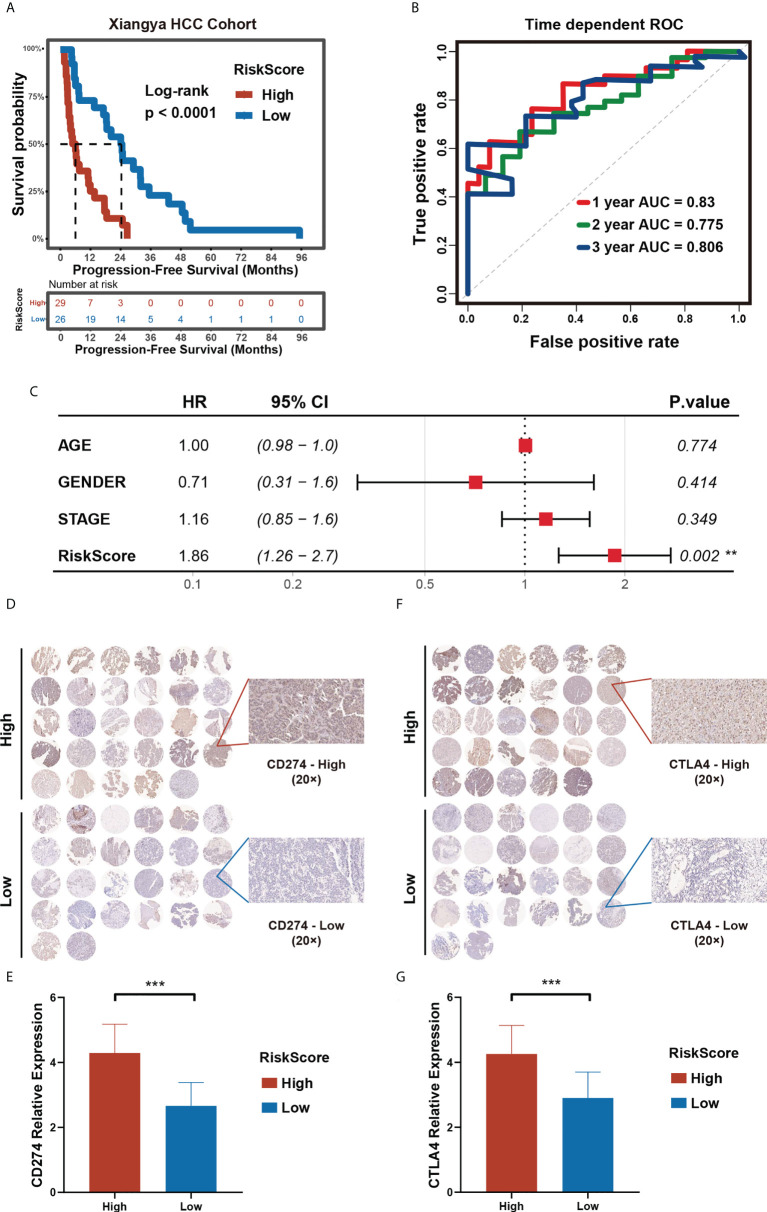
Validation of the prognostic risk model in Xiangya HCC cohort. **(A)** Progression-free survival time in high- and low- m^5^C score groups in Xiangya HCC cohort. **(B)** Time-dependent ROC curve (H) of the risk score in Xiangya HCC cohort. **(C)** Multivariate Cox regression analysis of PFS in Xiangya HCC cohort. **(D–G)** The expression level of CD274 **(D, E)** and CTLA4 **(F, G)** in high- and low- m^5^C score groups in the Xiangya HCC cohort. (*p < 0.05, **p < 0.01, ***p < 0.001, ns, no significant difference).

In the Xiangya HCC cohort (**Table S5**), the prognoses were approximately the same as those seen in the training set. Survival analysis verification testified that the prognostic risk score model was a great independent prognostic factor in HCC (**Figures 9A–C**). The expression of 6 candidate genes in HCC and paired normal liver tissues, CBX2, PBK, and KRT17 were higher in tumors than in normal tissues. On the contrary, FTCD, LCAT, and SOCS2 were lower in tumors than in normal tissues (**Figure S7**, *p*< 0.05).

To evaluate the predictive value of the prognostic risk score model on immunotherapy response, we selected CD274 and CTLA4, two immune checkpoints approved for clinical treatment, for further verification of the model. We collected pathological tissues from the 55 patients in the Xiangya HCC cohort. The tissues were used to make paraffin sections and for immunohistochemical staining. The results obtained show that higher CD274 and CTLA4 expression in the high prognostic risk score group compared to that of the low prognostic risk score group, a finding in line with the results of our bioinformatics analysis (**Figures 9D–G**). Our research provides further verifications that patients with a low prognostic risk score could benefit from immunotherapy to a higher degree than those with a high prognostic risk score. Hence, a prognostic risk score can be utilized as a potential biomarker for immunotherapy response.

## Discussion

Advanced HCC has a poor prognosis. There are some reasons behind it. Firstly, the diagnosis is often delayed. Many patients are diagnosed at an advanced stage. Then, due to the tumor heterogeneity, a large proportion of patients develop varying degrees of drug resistance. Immunotherapy, while powerful, does not work for everyone (41). Therefore, it is a critical challenge to detect early-stage HCC and find the best-personalized treatment in the field.

To our knowledge, this study is the first to (I) construct one prognostic and two diagnostic models based on comprehensive 18 m^5^C regulators; (II) build an m^5^C score to predict immunotherapy response, and (III) find the potential targeted drugs for patients who are less susceptible to ICBs. The past researches on m^5^C regulators-mediated methylation modification model in HCC need to be refined. This study used 18 m^5^C regulators to construct models that better improved the predictive accuracy of early diagnostic, immune contexture, and characterizations in HCC and validated the model by Xiangya real-world cohorts and three immunotherapy cohorts, and two TCGA digestive cancers cohorts to strengthen the persuasion. More importantly, our study represents a step toward individualized chemotherapy and immunotherapy for patients with HCC and provides new potential drugs for those ICB-insensitive patients.

6 candidate genes have been reported in HCC. It was reported that CBX2 overexpression occurs in a wide range of human tumors, including lung cancer, HCC, breast cancer, and so on. Previous studies presented that it could promote HCC progression *via* the phosphorylation of YAP (42). SOCS2 is reported to be highly expressed in HCC, associated with the N6-methyladenosine (m^6^A) in tumor pathogenesis and progression (43). According to past studies, LCAT (44) and FTCD (45) were expressed low, while PBK and KRT17 were overexpressed in HCC (46). All these findings were consistent with our results. We applied the prognostic model to immunotherapy and other GI tumor cohorts and found out that our prognostic risk model can distinguish between high and low-risk groups with a completely distinct OS or PFS in three immunotherapy cohorts, TCGA-CHOL, and TCGA-PAAD cohorts. These results indicate that our prognostic model has wider feasibility.

Most HCCs develop in stages, starting with chronic hepatitis, cirrhosis, and dysplastic nodules (DN) and ending with HCC. Radiological and pathological exams are the most common methods for early detection of HCC. Cirrhosis and tiny nodules, however, are becoming more difficult to be characterized, according to the latest findings (47). To circumvent these limitations, molecular markers that can objectively and precisely characterize HCC need to be identified. Two diagnostic models including SOCS2, LCAT, FTCD, KRT17, PBK, and CBX2 expression were proved to accurately separate HCC from normal and liver cirrhosis samples in this study. This result shows indications that the model can enhance the early detection rate of HCC and is beneficial to early clinical management in patients with HCC, therefore improving patients’ prognosis and lowering the risk of relapse. Our findings pave the way for the use of biomarkers such as SOCS2, LCAT, FTCD, KRT17, PBK, and CBX2 in the early detection of HCC patients.

The immune microenvironment provides strategies for immunotherapy and its characteristics are closely associated with immunotherapy efficacy (48). In our study, the high-m^5^C score group has a higher immune checkpoints expression level and a higher TMB, but a probably worse prognostic in immunotherapy response. These findings project a different opinion contrary to some previous studies and they demonstrate that the analysis of immune checkpoints or TMB only is insufficient to achieve accurate outcome prediction (49), just like we don’t have to test the immune checkpoints expression level and TMB before administrating to the patients with immunotherapy in all cancers according to clinical guidelines. The Tumor Immune Dysfunction and Exclusion (TIDE) algorithm is a wildly applied computational method to comprehensively evaluate the ICB therapy efficacy in tumors. Immunophenoscore (IPS) is of significant value in predicting response to anti-CTLA-4 and anti-PD-1 regimens, by quantifying the determinants of tumor immunogenicity and depicting the intratumoral immune landscapes and cancer antigenomes. The high-m^5^C score group has a higher TIDE and Exclusion score, MDSC score and lower Dysfunction score and IPS score. We could speculate that these patients in the high-m^5^C score group may have an immune evasion mechanism. Accordingly, patients with a low m^5^C score have a greater chance to benefit from ICB therapy.

Since patients in the high-m^5^C score group are insensitive to the ICB therapy, are there any other options for them? Greater attention should be paid to patients who are less susceptible to immunotherapy. By correlation analysis, the top 10 associated compounds with m^5^C scores in two drug databases are obtained. Although these 20 candidate compounds showed increased drug sensitivity in patients with higher m^5^C scores, the above analyses alone can not lead to the conclusion that these chemicals had a therapeutic effect on HCC. Thus, we used the CMap analysis to find compounds in which gene expression patterns were oppositional to the HCC-specific expression patterns. Secondly, we calculated fold-change differences in the mRNA expression levels of candidates’ drug target genes between tumor and normal tissue. Finally, a comprehensive literature search was performed in PubMed to find out the experimental and clinical evidence of candidate compounds for treating HCC. Four compounds, including Sepantronium bromide (YM-155), axitinib, vinblastine, and docetaxel, which had strong *in vitro* and silico evidence, were thought to be the most promising therapeutic candidates in HCC patients with high m^5^C scores. YM-155 is a small imidazolium-based agent that works as a surviving inhibitor. In HCC cell lines, YM155 could suppress proliferation and induce cell cycle arrest and apoptosis, giving rise to DNA damage by dysregulating genes related to cell-cycle checkpoints regulation. Moreover, in a mouse model, YM155 demonstrated significantly better efficacy than sorafenib. However, its therapeutic efficacy has only been moderate so far (50). By identifying potential YM155-responsive patients, the current study shed fresh light on how to improve the therapeutic efficacy of YM-155, and therefore on how to deliver precision medicine for HCCs. Axitinib is a potent and selective vascular endothelial growth factor receptors 1-3 inhibitor that has been extensively used as the first-line treatment in several anticancer regimens. In clinical trials, it contributes to remarkable longer PFS and TTP and a higher clinical benefit rate, with acceptable adverse effects in patients with advanced HCC (51). Gemcitabine and docetaxel for HCC seem to have potential benefits, as measured by OS, but their toxicity is an unignorable issue (52). Vinblastine has been widely known as a prominent agent in cancer chemotherapy. And it was reported to sustain antitumor activity by co-targeting mTOR and the microtubule in HCC (53). However, their association with HCC progression or m^5^C modification is still unknown. In addition, the high m^5^C score group has a lower AUC, which means that this group of patients is more sensitive to the predicted drugs. Since patients in the high-m^5^C score group have a poorer prognosis and are less likely to benefit from ICB therapy. We can presume that the predicted drugs may offer new options to patients in the high m^5^C score group who are less susceptible to immunotherapy.

This study investigates the expression level of 18 m^5^C regulators and survival data from TCGA. After univariate Cox regression and LASSO regression, a total of 6 candidate genes were obtained, including CBX2, SOCS2, LCAT, PBK, KRT17, and FTCD. Using the 6 candidate genes, 2 diagnostic and a prognostic models were established and they displayed a great predictive accuracy of early diagnostic, prognostic, immune contexture, and drug sensitivity in HCC. The prognostic model showed that patients with a high m^5^C score had a poorer prognosis and shorter survival time when compared to patients with a low m^5^C score. The AUC in the training set, validation set, and real-world cohort are most above 0.7. All of these results suggest that both the prognostic model and the diagnostic model are powerful predictive factors in HCC. In addition, the high m^5^C score group has a higher (I) inhibitory immune checkpoints expression level (such as IL-10、TGF-β、IL-4, and IL-35), (II) TIDE and Exclusion score (III) MDSC score, and a lower (I) Dysfunction score and (II) IPS score, which suggests that patients with a high m^5^C score have a higher chance to experience an immune evasion state and may have a worse response to immunotherapy. For further clinical translation, potential targeted drugs for high m^5^C score samples can be designed. Our research suggests that the diagnostic model plays a significant role in early diagnosis and the m^5^C score is an independent prognostic factor for HCC patients. Furthermore, the m^5^C score can serve as a potential biomarker that helps screen the best beneficiaries of immunotherapy and helps to find potential targeted drugs.

However, there are some limitations to our study. Firstly, due to the time constraints and budget limits, our research failed to thoroughly investigate the fundamental mechanism of m^5^C regulators involved in HCC, and further experimental and clinical validations are in need to facilitate the clinical application of our findings. Secondly, we were unable to directly assess the relationship between the m^5^C score and the response of HCC patients to immunotherapy because of the lack of overall clinical information in the datasets involved. Thirdly, the sample size of the Xiangya HCC cohort should be further enlarged in the future.

## Conclusion

To conclude, by integrating expression data from TCGA, GEO, and a real-world cohort, we successfully identified 6 candidate genes and constructed 2 diagnostic models that show great performance in early screening of HCC and an m^5^C score model that enable effective prediction of the prognosis of HCC patients. Patients with low m^5^C scores are more sensitive to ICBs and thus can experience a better life quality with a satisfactory prognosis. On the other hand, our study provided patients with high m^5^C scores with potential therapeutic targets and agents, which could significantly improve their prognosis. Overall, this research has shed fresh light on individualized prognostic methods as well as the integration of tailored prognosis prediction for precision medicine.

## Data availability statement

The datasets presented in this study can be found in online repositories. The names of the repository/repositories and accession number(s) can be found in the article/**Supplementary Material**.

## Ethics statement

The studies involving human participants were reviewed and approved by the Xiangya Hospital Medical Ethics Committee of Central South University. The patients/participants provided their written informed consent to participate in this study.

## Author contributions

WL, ZF and HS supervised the study. PL, WL, ZF and HS designed the study. ES, SZ, PL, YHL and WL drafted the manuscript. LG, YL, ZJ and WW did the statistical analysis. CG, YP, YG, YTL and QH analyzed and interpreted the data. ZZ, JM, LW, YH, XT, YC and CC collected the data and performed the major analysis and experiments. All authors contributed to the article and approved the submitted version.

## Funding

This study was supported by grants from the National Key R & D Program of China (No. 2018YFC1313300), National Natural Science Foundation of China (No.: 81070362, 81172470, 81372629, 81772627, 81874073 & 81974384), key projects from the Nature Science Foundation of Hunan Province (No. 2015JC3021 & 2016JC2037), the projects from Beijing CSCO Clinical Oncology Research Foundation (No. Y-HR2019-0182, Y-2019Genecast-043).

## Acknowledgments

The authors would like to give their sincere appreciation to the reviewers for their helpful comments on this article and research groups for the TCGA and CEO, which provided data for this collection, and the included patients and their family members.

## Conflict of interest

The authors declare that the research was conducted in the absence of any commercial or financial relationships that could be construed as a potential conflict of interest.

## Publisher’s note

All claims expressed in this article are solely those of the authors and do not necessarily represent those of their affiliated organizations, or those of the publisher, the editors and the reviewers. Any product that may be evaluated in this article, or claim that may be made by its manufacturer, is not guaranteed or endorsed by the publisher.

## Supplementary material

Supplementary Figure 1 The CNV of m5C regulator genes in TCGA-LIHC.

Supplementary Figure 2 TClustering of HCC based on 18 m5C regulators. (A). The consensus score matrix of all samples when k = 2. A higher consensus score between two samples indicates they are more likely to be grouped into the same cluster in different iterations. (B). The cumulative distribution functions of the consensus matrix for each k (indicated by colors). (C-D). The best number of clusters k = 2, calculated by Silhouette algorithm (C) and R package “NbClust” (D). (E). PCA plot by the expression of 18m5C regulator genes in the two m5C clusters. The blue dots represented Cluster1, and the red dots represented Cluster2. (F). tSNE plot by the expression of 18 m5C regulator genes in two m5C clusters. The blue dots represented Cluster1, and the red dots represented Cluster2. (G, H) Kaplan-Meier analysis for OS (G) and PFS (H) of two m5C clusters in the TCGA-LIHC cohort.

Supplementary Figure 3 Multivariate Cox analysis of training cohort TCGA-LIHC.

Supplementary Figure 4 (A, B) Kaplan-Meier analysis for OS (A) and time-dependent ROC curve (B) of the risk score in the GSE14520 cohort. (C, D) Kaplan-Meier analysis for RFS (C) and time-dependent ROC curve (D) of the risk score in the GSE14520 cohort.

Supplementary Figure 5 Immune cell infiltration by CIBERSORT(A), TIMER(B) and MCPcounter(C) between high- and low-m^5^C score groups.

Supplementary Figure 6 Immunophenoscore (IPS) between high- and low- m^5^C score groups.

Supplementary Figure 7 Validation of risk score in immunotherapy cohorts and other TCGA digestive cancers. (A-H) Kaplan-Meier curves for patients with high- and low- m5C scores in three immunotherapy cohorts IMvigor210 cohort (A), Riaz et al., Cell 2017 (B), Lauss et al., Nat Commun 2017 (C), and other TCGA digestive cancer cohorts included TCGA-PAAD (D), TCGA-CHOL (E), TCGA-STAD (F), TCGA-COAD (G), and TCGA-READ (H).

Supplementary Figure 8 Differential expression of six selected genes in HCC and adjacent non tumor liver tissues in Xiangya HCC cohort.
