# Supplementary figures and images for: Prognostic stratification based on m5C regulators acts as a novel biomarker for immunotherapy in hepatocellular carcinoma
Source: Front Immunol. 2022 Sep 9;13:951529. doi: 10.3389/fimmu.2022.951529 (PMC9505913; doi:10.3389/fimmu.2022.951529)

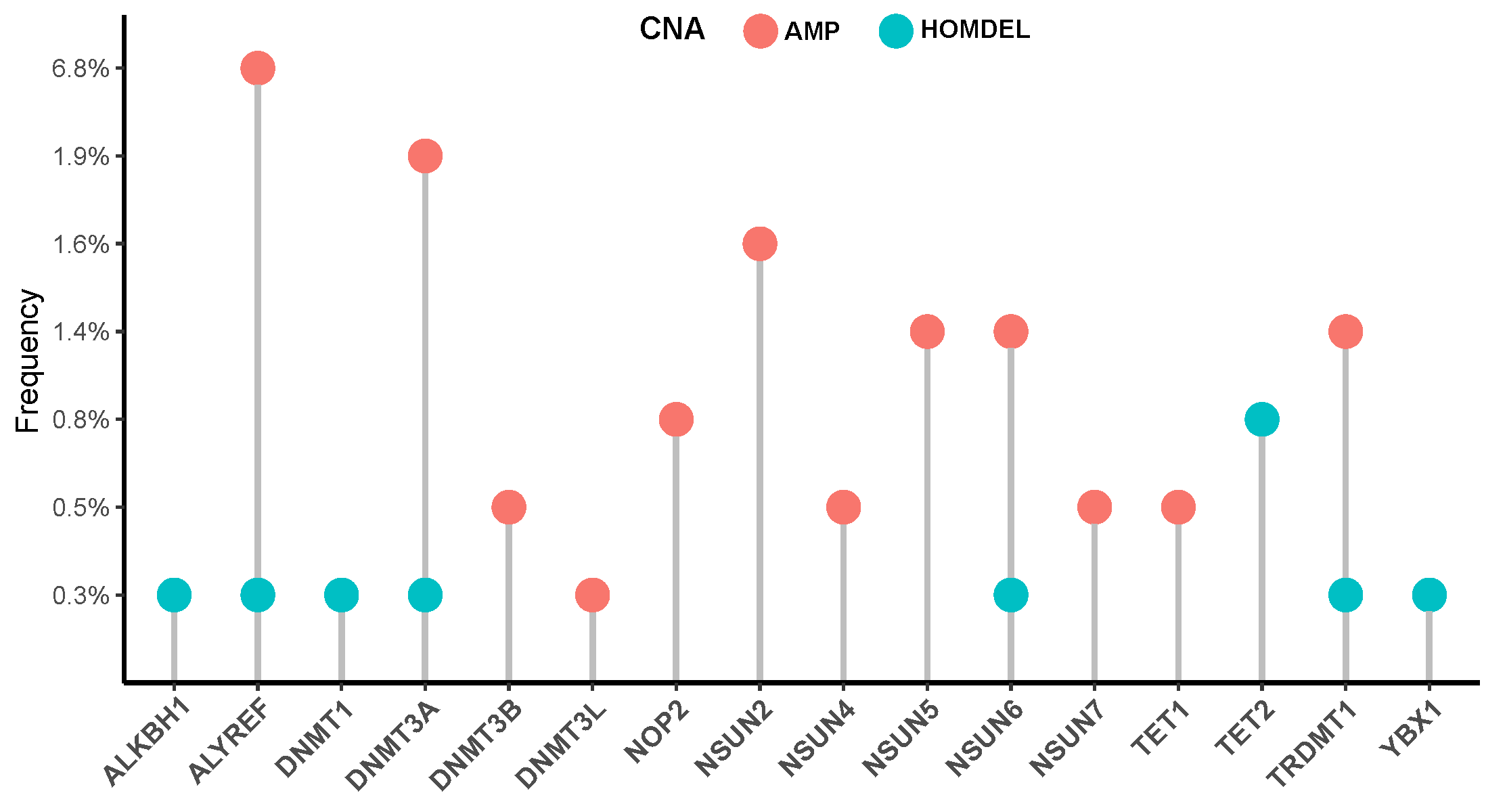

Supplement: Supplementary Figure 1 — The CNV of m5C regulator genes in TCGA-LIHC. [file Image_1.tif]

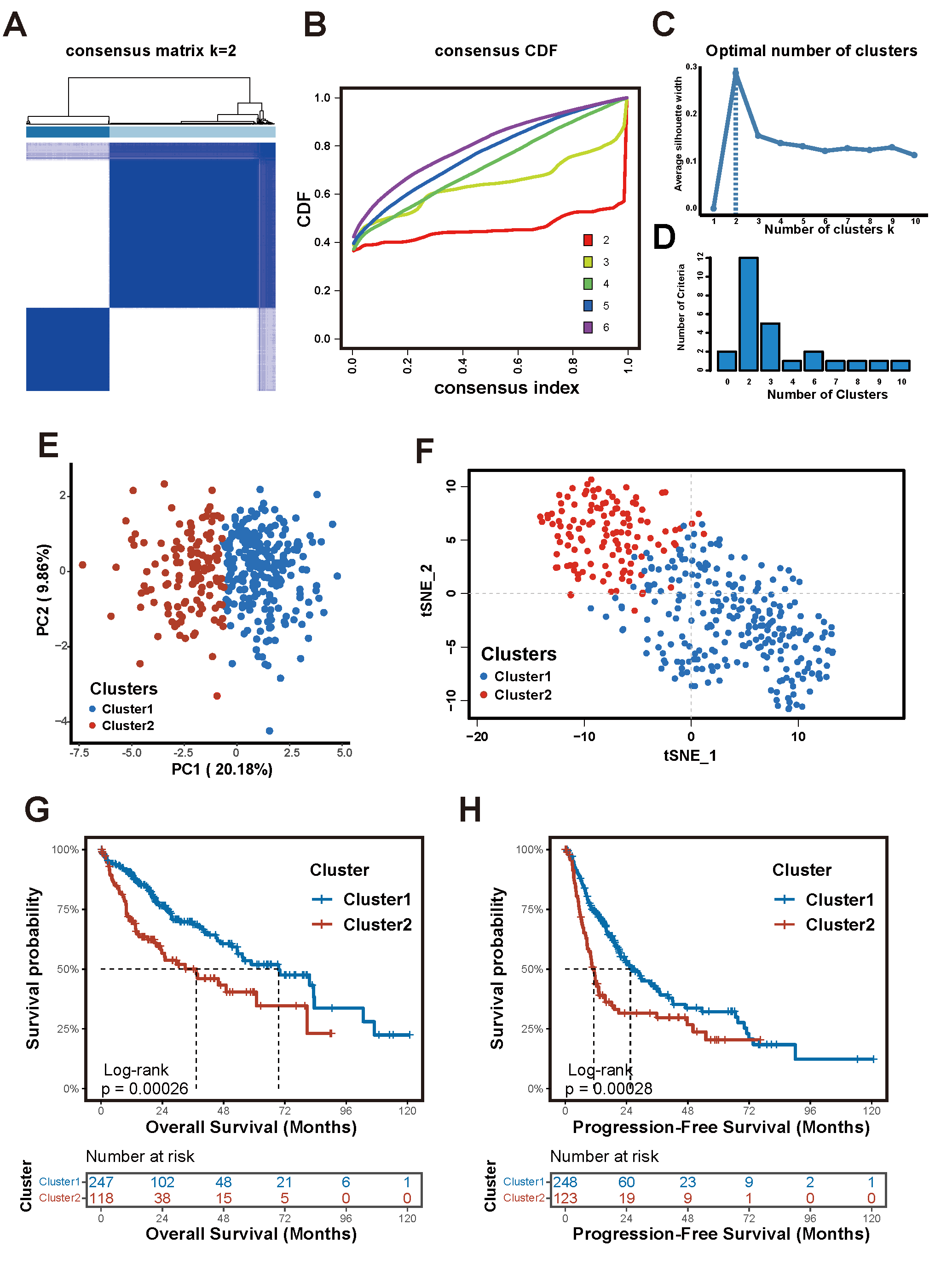

Supplement: Supplementary Figure 2 — TClustering of HCC based on 18 m5C regulators. (A). The consensus score matrix of all samples when k = 2. A higher consensus score between two samples indicates they are more likely to be grouped into the same cluster in different iterations. (B). The cumulative distribution functions of the consensus matrix for each k (indicated by colors). (C-D). The best number of clusters k = 2, calculated by Silhouette algorithm (C) and R package “NbClust” (D). (E). PCA plot by the expression of 18m5C regulator genes in the two m5C clusters. The blue dots represented Cluster1, and the red dots represented Cluster2. (F). tSNE plot by the expression of 18 m5C regulator genes in two m5C clusters. The blue dots represented Cluster1, and the red dots represented Cluster2. (G, H) Kaplan-Meier analysis for OS (G) and PFS (H) of two m5C clusters in the TCGA-LIHC cohort. [file Image_2.tif]

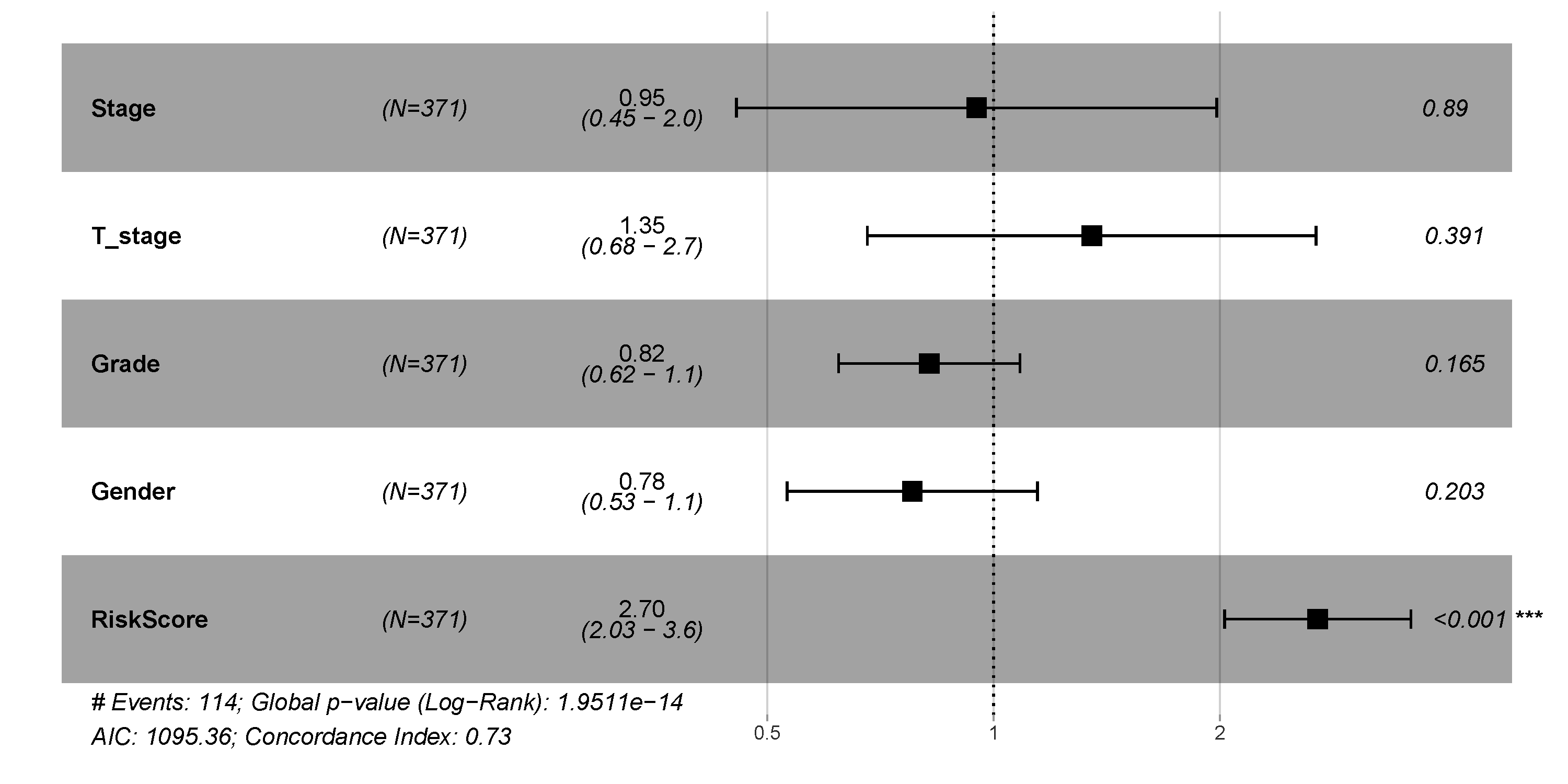

Supplement: Supplementary Figure 3 — Multivariate Cox analysis of training cohort TCGA-LIHC. [file Image_3.tif]

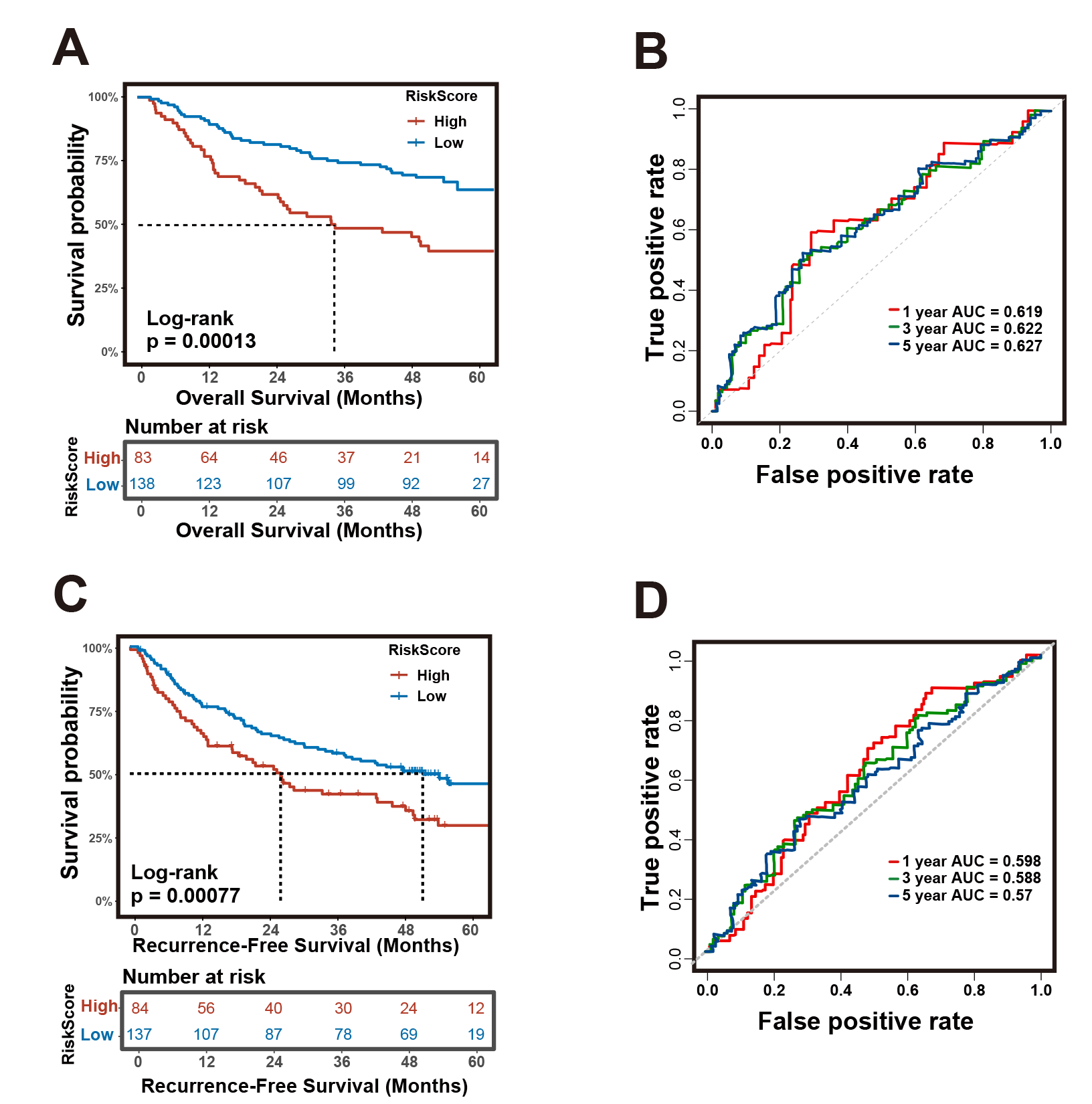

Supplement: Supplementary Figure 4 — (A, B) Kaplan-Meier analysis for OS (A) and time-dependent ROC curve (B) of the risk score in the GSE14520 cohort. (C, D) Kaplan-Meier analysis for RFS (C) and time-dependent ROC curve (D) of the risk score in the GSE14520 cohort. [file Image_4.tif]

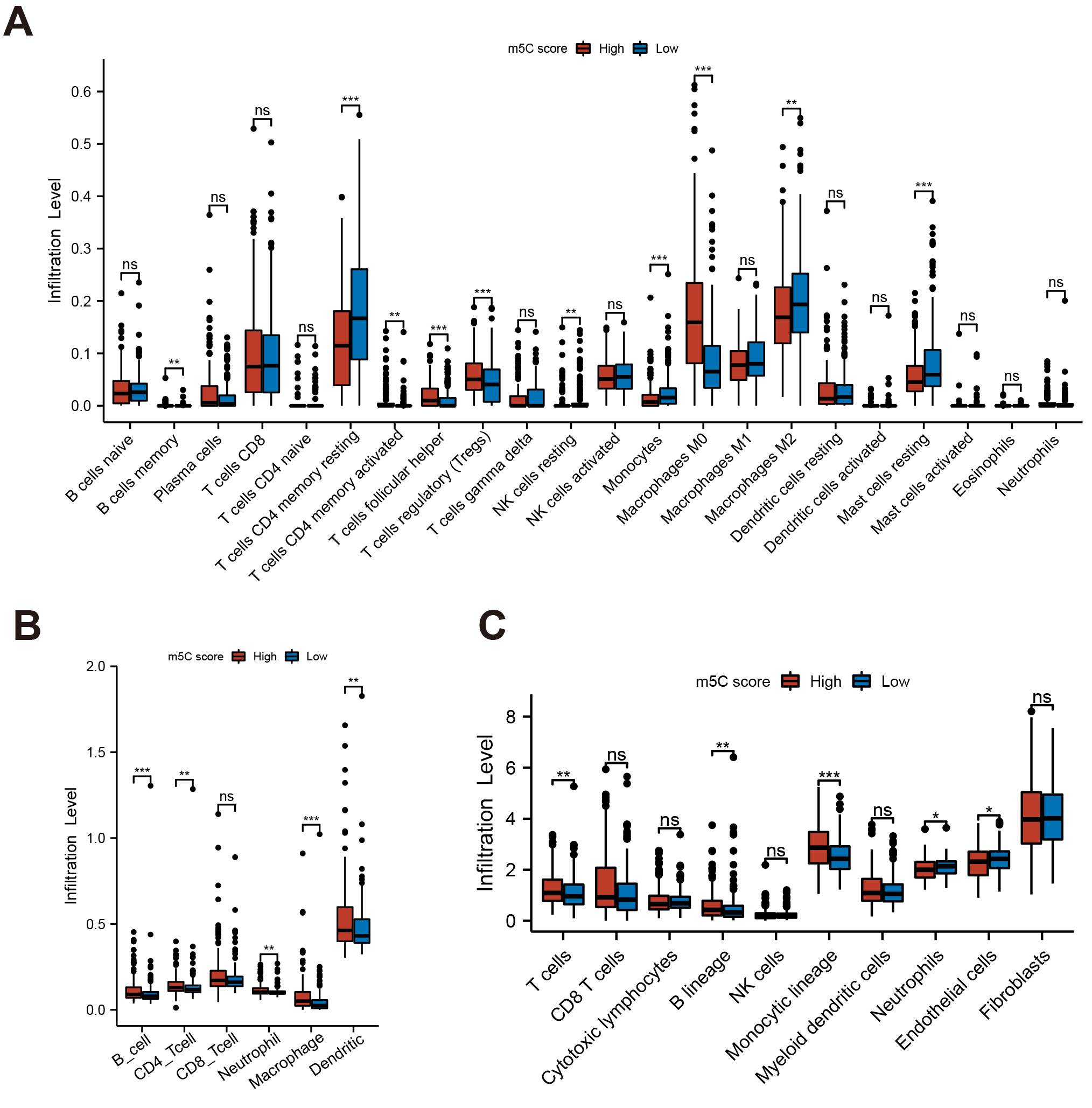

Supplement: Supplementary Figure 5 — Immune cell infiltration by CIBERSORT(A), TIMER(B) and MCPcounter(C) between high- and low-m5C score groups. [file Image_5.tif]

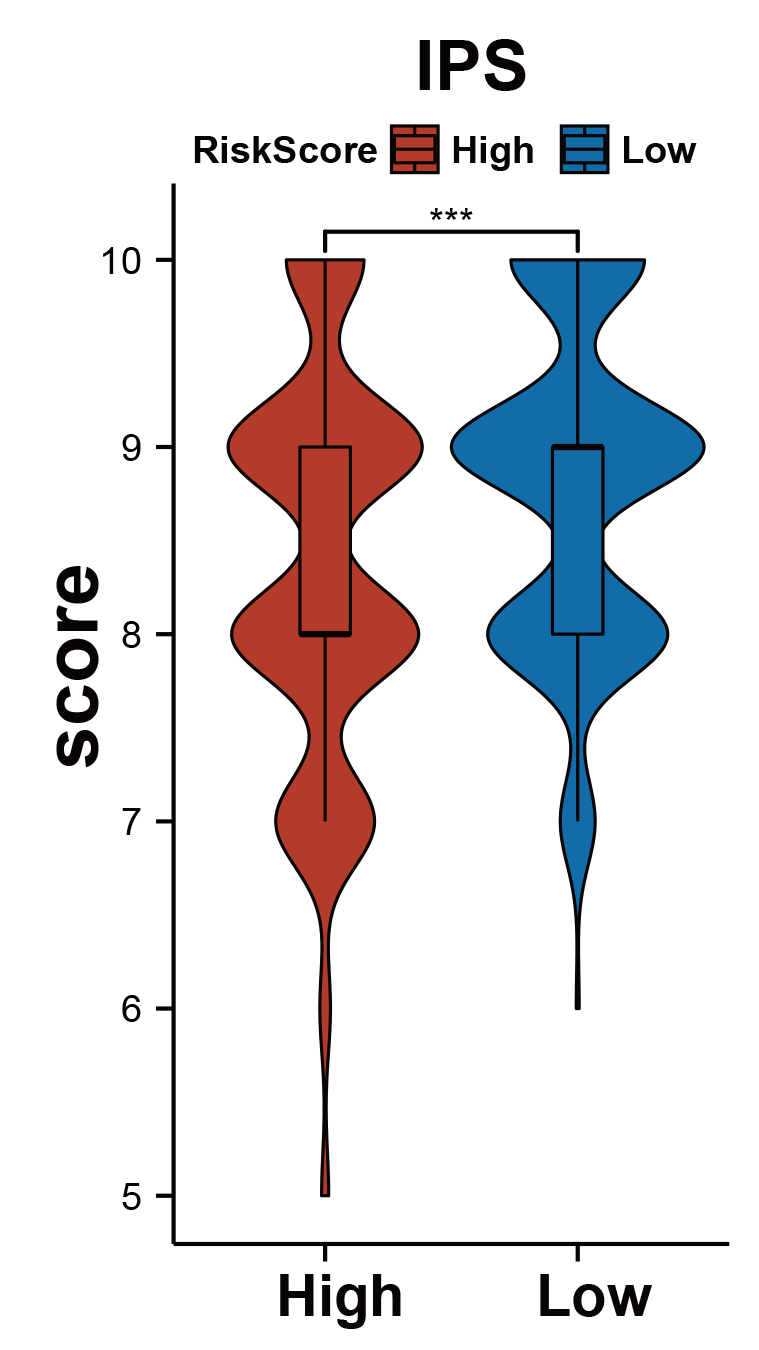

Supplement: Supplementary Figure 6 — Immunophenoscore (IPS) between high- and low- m5C score groups. [file Image_6.tif]

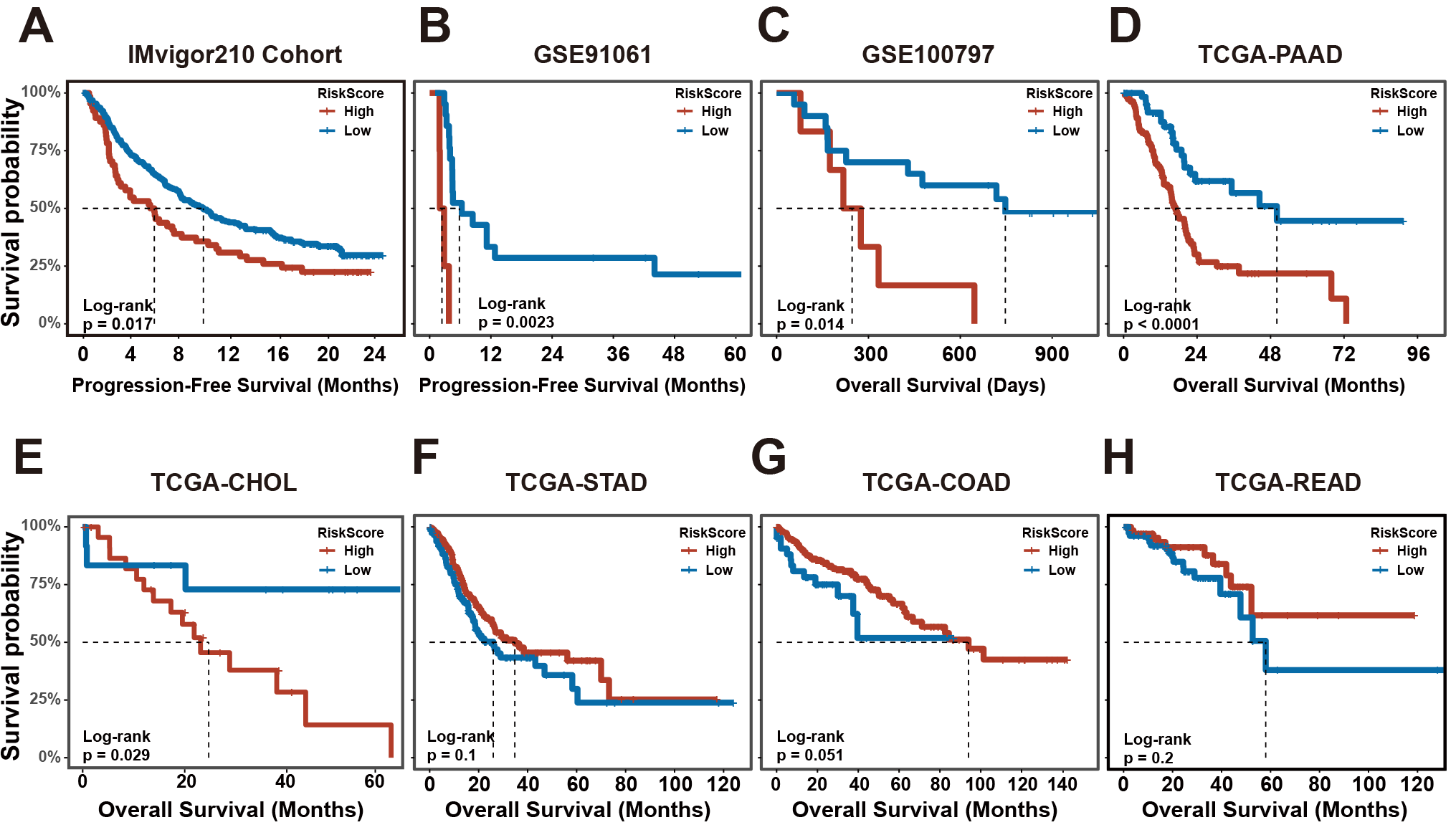

Supplement: Supplementary Figure 7 — Validation of risk score in immunotherapy cohorts and other TCGA digestive cancers. (A-H) Kaplan-Meier curves for patients with high- and low- m5C scores in three immunotherapy cohorts IMvigor210 cohort (A), Riaz et al., Cell 2017 (B), Lauss et al., Nat Commun 2017 (C), and other TCGA digestive cancer cohorts included TCGA-PAAD (D), TCGA-CHOL (E), TCGA-STAD (F), TCGA-COAD (G), and TCGA-READ (H). [file Image_7.tif]

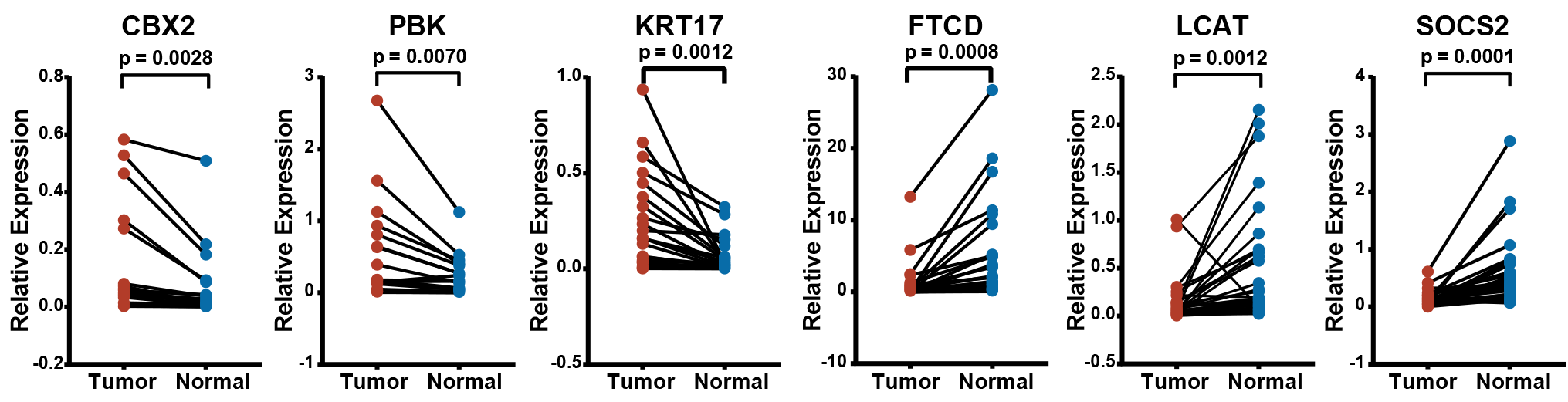

Supplement: Supplementary Figure 8 — Differential expression of six selected genes in HCC and adjacent non tumor liver tissues in Xiangya HCC cohort. [file Image_8.tif]
